# An Apology

**Published:** 1883-05

**Authors:** 


### AN APOLOGY.

We are sure that when our readers come to know the circumstances under which the April and May numbers of this journal were issued, they will forgive all editorial deficiencies, and pardon the delay in their reception. While we are at it we might as well clear our conscience completely, and ask indulgence for a meditated fault. The June number will not be sent out until about the middle of the month, but we hope that when all contemplated changes shall have been made, there will not again occur a necessity for this most ungracious of tasks, the apology for necessary delay. For the dearth of editorial matter in this number we are not at all sure that any excuse will be demanded, but if it should be, we must plead the unusual extra duties imposed upon us, and offer in expiation the two leading original communications, which all will agree have sufficient merit to atone for any editorial shortcomings.

